# The Spinocerebellar Ataxia 34-Causing W246G ELOVL4 Mutation Does Not Alter Cerebellar Neuron Populations in a Rat Model

**DOI:** 10.1007/s12311-024-01708-8

**Published:** 2024-06-08

**Authors:** Jennifer L. Fessler, Megan A. Stiles, Martin-Paul Agbaga, Mohiuddin Ahmad, David M. Sherry

**Affiliations:** 1 Department of Cell Biology, University of Oklahoma Health Sciences Center, 940 S.L. Young Blvd, BMSB-100, Oklahoma City, OK 73104 United States of America; 2 Department of Ophthalmology, Dean McGee Eye Institute, Oklahoma City, OK 73104 United States of America; 3 Department of Neurosurgery, University of Oklahoma Health Sciences Center, Oklahoma City, OK 73104 United States of America; 4 Department of Pharmaceutical Sciences, University of Oklahoma Health Sciences Center, Oklahoma City, OK 73104 United States of America

**Keywords:** SCA34, Purkinje cell, Granule cell, Molecular layer interneuron, Unipolar brush cell, Neurodegeneration, Neurodevelopment

## Abstract

Spinocerebellar ataxia 34 (SCA34) is an autosomal dominant disease that arises from point mutations in the fatty acid elongase, Elongation of Very Long Chain Fatty Acids 4 (ELOVL4), which is essential for the synthesis of Very Long Chain-Saturated Fatty Acids (VLC-SFA) and Very Long Chain-Polyunsaturated Fatty Acids (VLC-PUFA) (28–34 carbons long). SCA34 is considered a neurodegenerative disease. However, a novel rat model of SCA34 (SCA34-KI rat) with knock-in of the W246G ELOVL4 mutation that causes human SCA34 shows early motor impairment and aberrant synaptic transmission and plasticity without overt neurodegeneration. ELOVL4 is expressed in neurogenic regions of the developing brain, is implicated in cell cycle regulation, and ELOVL4 mutations that cause neuroichthyosis lead to developmental brain malformation, suggesting that aberrant neuron generation due to ELOVL4 mutations might contribute to SCA34. To test whether W246G ELOVL4 altered neuronal generation or survival in the cerebellum, we compared the numbers of Purkinje cells, unipolar brush cells, molecular layer interneurons, granule and displaced granule cells in the cerebellum of wildtype, heterozygous, and homozygous SCA34-KI rats at four months of age, when motor impairment is already present. An unbiased, semi-automated method based on Cellpose 2.0 and ImageJ was used to quantify neuronal populations in cerebellar sections immunolabeled for known neuron-specific markers. Neuronal populations and cortical structure were unaffected by the W246G ELOVL4 mutation by four months of age, a time when synaptic and motor dysfunction are already present, suggesting that SCA34 pathology originates from synaptic dysfunction due to VLC-SFA deficiency, rather than aberrant neuronal production or neurodegeneration.

## Introduction

Elongation of Very Long Chain Fatty Acids 4 (ELOVL4) is one of a family of seven mammalian ELOVL enzymes that are essential for the elongation of fatty acids to lengths greater than 16 carbons [1]. ELOVL4 elongates long chain saturated fatty acids and polyunsaturated fatty acids (SFA and PUFA) precursors of 24–26 carbon length to generate Very Long Chain-SFA and Very Long Chain-PUFA (VLC-SFA and VLC-PUFA, respectively) [1–3]. ELOVL4 is the only member of the ELOVL family that performs this function [3]. ELOVL4 expression is limited to the brain, retina, meibomian gland, testis, and skin [1, 4–9]. All cerebellar neurons express ELOVL4, with the highest expression in granule cells [1, 7].

The function of very long chain-fatty acids (VLC-FA) is tissue specific. In the brain, VLC-SFA are incorporated into complex sphingolipids and are enriched in synaptic vesicles [13]. VLC-SFA deficiency disrupts synaptic transmission in hippocampus, cerebellum, and retina [10, 13–15]. VLC-PUFA are not detectable in the healthy brain, after development [16]. In the retina, ELOVL4 generates mainly VLC-PUFA, which are packaged into phosphatidylcholine and incorporated into disks of the photoreceptor outer segment [8, 9, 16]. In the skin and Meibomian gland, VLC-SFA are packaged into ω-O-acyl-ceramides and are essential for forming the water barrier in the skin and tear film [5, 6].

Three different sets of mutations in *ELOVL4* cause three distinct neurodegenerative diseases: Spinocerebellar Ataxia 34 (SCA34), Stargardt’s Macular Dystrophy (STGD3), and neuroichthyosis [1]. Spinocerebellar ataxia (SCA) is an autosomal dominant disorder that affects roughly 3 in 100,000 people [17]. Over 40 different types of SCA have been identified and arise from a wide range of mutations. All forms of SCA are characterized by ataxia and cerebellar atrophy, but additional neurological symptoms also may be present [17, 18].

Spinocerebellar ataxia 34 with or without erythrokeratosis variabilis (EKV) arises from several *ELOVL4* variants with single nucleotide polymorphisms in *ELOVL4* that generate a full length protein with single amino acid substitutions. Six distinct *ELOVL4* variants that cause SCA34 have been identified [19–25]. In addition to ataxia and cerebellar atrophy, SCA34 patients often show dysarthria, apraxia, nystagmus, and EKV. Typical onset of SCA34 is between the 20s to the 60s, depending on the specific *ELOVL4* variant. Recently, cognitive impairments have been recognized in some patients [26]. Patients with the c.512T > C, (p.I171T) ELOVL4 variant show ataxia that may appear together with retinitis pigmentosa with or without EKV [27]. Another ELOVL4 variant (L168S) causes both retinal and SCA34 pathologies [24].

Two other sets of *ELOVL4* variants cause neurological diseases distinct from SCA34. Autosomal dominant inheritance of *ELOVL4* nonsense mutations cause Stargardt’s Macular Dystrophy (STGD3), an aggressive form of macular dystrophy that causes early onset photoreceptor degeneration and blindness by the early twenties [28–32]. These mutations lead to early termination of the ELOVL4 protein and loss of the C-terminus endoplasmic reticulum targeting signal needed for retention in the endoplasmic reticulum [33]. Patients with STGD3 do not display ataxia or other neurological features. Neuroichthyosis is caused by homozygous recessive mutations in *ELOVL4* that cause early truncation of the ELOVL4 protein disrupts the transmembrane domains and its enzymatic catalytic core, rendering the protein inactive, causing neuroichthyosis [34–36]. Children with neuroichthyosis have severe seizures, intellectual disability, spastic quadriplegia, ichthyosis, and early death [34–36].

To better understand the role of ELOVL4 and its VLC-FA products in the brain, we generated a knock-in rat model of SCA34 (SCA34-KI rat) with insertion of the c.736T > G (p. W246G) *ELOVL4* mutation that causes SCA34 using CRISPR-Cas9 gene editing [12, 37]. ELOVL4 expression in the brain of heterozygous (HET) or homozygous (MUT) SCA34-KI rats is comparable to wildtype (WT) rats [12]. Lipidomic analysis of skin and retina of SCA34-KI rats showed that W246G ELOVL4 selectively impairs VLC-SFA synthesis but did not affect VLC-PUFA synthesis [37]. Consistent with this finding, other SCA34-causing ELOVL4 variants also selectively impair VLC-SFA synthesis [24, 38]. Both HET and MUT SCA34-KI rats show motor impairment by two months of age [37]. Impairment of synaptic plasticity, synapse-specific defects in synaptic transmission, and reduced numbers of dendritic spines on Purkinje cells are all evident in MUT SCA34-KI rats by four months of age [15]. However, no evidence of overt neurodegeneration, disruption of cortical layering or thickness or loss of Purkinje cells is observed in SCA34-KI rats up to six months of age [12].

Spinocerebellar ataxia 34 is considered to be a neurodegenerative disease, as degeneration of the cerebellum is the clinical hallmark of the disease in human patients [19–25]. However, recent studies in the SCA34-KI rat suggest that other pathological mechanisms may be present before the onset of clinically recognized symptoms and neurodegeneration. Although it is clear that aberrant VLC-SFA metabolism is a key element in the development of SCA34 [14, 24, 38], the mechanisms by which SCA34 arises from ELOVL4 mutation are poorly understood. One potential mechanism by which ELOVL4 mutations could affect the cerebellum is by altering the production or balance of neurons during cerebellar development. ELOVL4 is highly expressed in neurogenic regions of the developing brain, including the fourth ventricle [7]. ELOVL4 also has been linked to ciliary signaling, which regulates cell cycle and cell production [39]. Patients with neuroichthyosis arising from ELOVL4 mutations also show malformation of the brain, suggesting aberrant cell production, survival, and or/migration during brain development [34]. To test whether the SCA34-causing W246G ELOVL4 mutation altered the numbers or balance of specific classes of cerebellar neurons, quantitative immunolabeling approaches were used in the cerebellum of WT, HET, and MUT SCA34-KI rats at four months of age, a time point when cerebellar function is impaired in the absence of overt neurodegeneration.

## Methods

### Animals

The SCA34-KI rat model was generated on the Long-Evans background using CRISPER/Cas9 to knock-in the c.736T > G (p.W246G) mutation in *Elovl4* that causes human SCA34 [20] as previously described [14, 37]. Rats were kept on a 12 h light:12 h dark cycle (light intensity of 25–40 lx at cage level) with food and water available *ad libitum*. All rats used in these studies were 120 days of age. Animal procedures were approved by the Institutional Animal Care and Use Committee of the University of Oklahoma Health Sciences Center and met National Institute of Health guidelines.

#### Tissue Preparation

Rats were anesthetized using ketamine (100 mg/kg body weight) and xylazine (5 mg/kg body weight), then perfused using 4% paraformaldehyde in 0.1 M phosphate buffered saline (PBS) through the left ventricle as described previously [12]. The brain was harvested and fixed in 4% PFA at 4 °C for an additional seven days. The brain was rinsed thoroughly with PBS, and stored in PBS until use. To prepare tissue sections, the cerebellum was isolated and hemisected along the midline, embedded in 7% agarose gel, and sectioned in the sagittal plane using a vibratome at 40–60 μm thick. All sections from within a given specimen were cut at the same thickness, based on the optimum for that specimen, and were stored in 1x PBS at 4˚C for up to 3 months (in the absence of preservatives). There was no systematic selection of sections for immunolabeling.

#### Analysis of Cortical Layers and Purkinje Cell Density

**Fig. 1:**
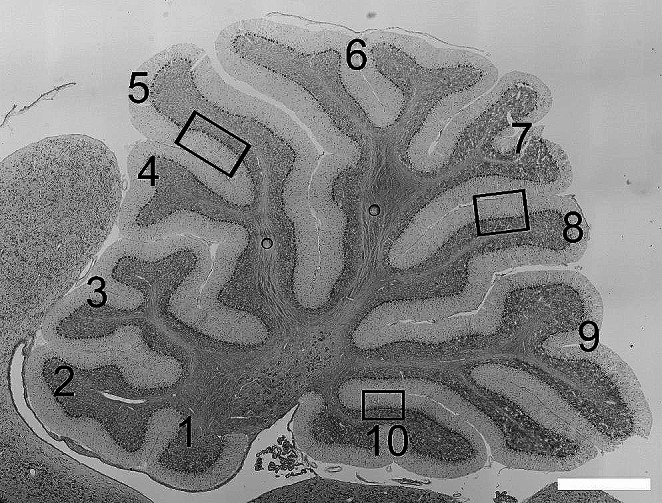
Sampling for quantitative analyses in sagittal cerebellar sections. boxes indicate the regions in lobules 5, 8, and 10 sampled for cell population analysis. Scale bar: 1 mm

Free-floating vibratome sections were stained with 0.1 M Toluidine Blue for 10 min on a slide warmer at 59˚C. Sections were rinsed in PBS at room temperature with gentle agitation, then cover-slipped using Mowiol or Prolong Gold. Image montages of stained cerebellar sections were acquired using a Leica M205-MFC or a Nikon Ti2 inverted microscope empirically calibrated for scale. The width of the various layers of the cerebellar cortex were measured manually using ImageJ software as described previously [12]. To ensure consistent sampling, images of cerebellar cortex were acquired on linear portions of lobule 5 (inferior side of the lobule), lobule 8 (superior side of the lobule), and lobule 10 (superior side of the lobule) (Fig. 1). A line perpendicular to the white matter was drawn across each cortical layer to measure width. Linear density of Purkinje cells (PCs) in the Purkinje Cell Layer (PCL) was calculated by counting the number of PCs and dividing the number of cells by length of the region of the PCL counted [12]. Analysis was performed using well-oriented sections displaying Purkinje cells in a discrete monolayer to prevent any potential miscounting of Purkinje cells. These analyses were performed using sections prepared from female rats.

#### Immunohistochemistry

Sagittal vibratome sections were rinsed in 1x PBS, then incubated for 2 h at room temperature in blocker (10% Normal Goat Serum, 5% Bovine Serum Albumin, and 0.5% Triton in 1x PBS). Well established primary marker antibodies for labeling of specific cerebellar cell populations (see details in Table 1) were diluted in blocker. Nuclei were labeled using DAPI (5 mg/ml) applied together with the primary antibodies. Sections were incubated in primary antibodies in a humidified container for seven days at room temperature. Sections were rinsed in 1x PBS, then incubated in appropriate secondary antibodies (Table 1) for two hours at room temperature, then rinsed in 1x PBS with gentle agitation. Sections were mounted onto glass slides in Mowiol for imaging. All immunolabeling combinations were performed in all animals whenever possible, however, in some cases there were not sufficient sections available to perform all possible labeling combinations.

**Table 1:** Antibodies

Antibody	Dilution	Company	Catalog #	RRID
NeuN (Mouse monoclonal)	1:500	Sigma/Millipore	MAB377, clone A60	AB_2298772
FOX2 (Chicken polyclonal)	1:2000	EnCor Biotechnology	CPCA-FOX2	AB_2744538
Calretinin (Rabbit polyclonal)	1:500	Millipore	AB5054	AB_2068506
Goat anti-Mouse IgG AlexaFluor488 (Polyclonal)	1:200	Thermo/Molecular Probes	A-11,008	AB_143165
Alexa Fluor 488-AffiniPure Fab Fragment Goat Anti-Mouse IgG (Polyclonal)	1:200	Jackson ImmunoResearch Labs	115-547-003	AB_2338869
Goat anti-Chicken IgY AlexaFluor 568 (Polyclonal)	1:200	Thermo Fisher Scientific	A-11,041	AB_2534098
Rhodamine Red-X-AffiniPure Fab Fragment (Polyclonal)	1:200	Jackson ImmunoResearch Labs	103-297-008	AB_2632422
Alexa Fluor 594-AffiniPure Fab Fragment Goat Anti-Chicken IgY (IgG), Fc Fragment Specific (Polyclonal)	1:200	Jackson ImmunoResearch Labs	103-587-003	AB_2632425
Goat anti-Rabbit IgG (H + L) Alexa Fluor 647 (Polyclonal)	1:200	Invitrogen	A32733	AB_2633282
Alexa Fluor 647-AffiniPure Fab Fragment Goat Anti-Rabbit IgG (H + L) (Polyclonal)	1:200	Jackson ImmunoResearch Labs	111-607-003	AB_2338084

### Workflow for Neuronal Quantification

**Fig. 2:**
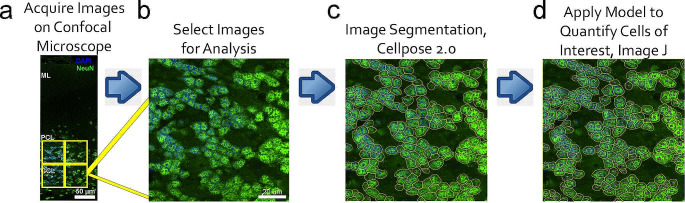
Workflow for cell quantitation. **A**. Images of neurons immunolabeled for cell-specific markers are obtained by confocal microscopy. **B**. Images are selected for analysis. Density of the immunolabeled cells of interest determines whether an entire cortical layer can be analyzed in a single image, or if analysis of a layer must be performed using multiple regions that encompass the entire layer. **C**. Multiple images are used to train a Cellpose 2.0 model to recognize the cells of interest. Once trained, the model is applied to the remaining images in the dataset. The trained model directs subsequent image segmentation to identify cells of interest or regions of interest (ROI), which are marked individually as a ROI that can be recognized by Image J. D. Following identification of individual cells of interest as independent ROIs in Cellpose 2.0, images are imported into Image J, and Image J is used to count the number of cells of interest (ROIs) in each image

Quantitative analysis of neuronal populations in WT, HET, and MUT rat cerebellum was based on a computer-assisted workflow to identify specific types of cerebellar neurons using immunolabeling for known markers for specific types of cerebellar neurons followed by confocal imaging, computer-assisted image segmentation, and cell counting (Fig. 2). Quantitation for each cell-specific marker was performed on minimum of 27 images from each animal in the analysis.

#### Image Acquisition

To minimize any effects of tissue laterality, all analyses were performed using sections obtained from the cerebellar vermis (between 0 and 1800 μm from the cerebellar midline). Sections for immunolabeling were selected at random from within this range to avoid any systematic bias. Sampling was performed in linear regions of lobules 5, 8, and 10 as described above (Fig. 1). Images were acquired from well-labeled sections using a Nikon W1-CSU Dual Spinning Disk Confocal microscope fitted with a Hamamatsu Orca Fusion BT sCMOS camera. Images of immunolabeling were collected as Z-stacks of 12 μm total depth at 0.3 μm step size, using a 60x objective lens with 1.4 numerical aperture. Image resolution was 0.10833 μm/px.

#### Image Preparation for Quantification

Due to the intrinsic differences in the density and location of the various types of cerebellar neurons in the layers of the cerebellar cortex, cell type-specific quantification required selection of regions of interest for cell counting from single X-Y image planes. Analysis was performed from multiple, non-overlapping Z-planes from within each image stack to prevent repeated sampling of individual cells. For granule cells, which are packed at very high density in the granule cell layer (GCL), images of labeling for NeuN and DAPI in the granule cell layer were cropped into 100 μm by 100 μm tiles for analysis. For analysis of molecular layer interneurons (MLIs) and displaced granule cells, which are present at low density in the molecular layer (ML), tiles spanning the entire width of the molecular layer were cropped from specimens labeled for FOX2, NeuN, and DAPI. For analysis of unipolar brush cells (UBCs), which are present at low to moderate density in the GCL, tiles spanning the entire width of the GCL were selected for analysis from specimens labeled for Calretinin and DAPI.

#### Selection of Image Sample Planes and Image Curation

An ImageJ macro was developed to select three non-overlapping image planes from each Z-stack for analysis: a plane from the top of the Z-stack, a plane from the middle of the Z-stack, and a plane from the bottom of the Z-stack, to prevent any repeated counting of individual cells. Out of focus image planes were excluded from analysis. Image tiles for analysis of GCs with less than 80% of the image area filled with GCs were excluded, to prevent under-sampling of GCs.

#### Model Training for Cell Quantification Using Cellpose 2.0

Cellpose 2.0 is a semi-supervised machine learning algorithm for image segmentation through an iterative human-machine training process [40]. A cell-specific model for cell counting that required colocalization of neuron-specific marker labeling and nuclear DAPI labeling was developed for each type of neuron to ensure that all objects counted were genuine neuronal cell bodies, not fragments or processes of a cell. Accordingly, training the model for each cell type required a different number of training images to achieve accurate identification of the cell of interest. Training the GC model required 50 + images; the MLI model required 30 + images; the displaced granule cell model, required 60 + images; and the UBC model required 150 + images. A criterion of at least five cells of interest within the image tile was required for an image to be included in any model.

#### Image Segmentation

The cell-specific segmentation model was applied to each image in a data set using Cellpose 2.0 and a python script created by the developers of Cellpose 2.0 and run on Jupyter Notebook [40]. The developers of Cellpose 2.0 also provided an Image J macro to create ROI.zip files for each image analyzed by Cellpose 2.0 (available at: https://github.com/MouseLand/cellpose/blob/main/imagej_roi_converter.py [40].

#### Quantification

To quantify the numbers of each type of cerebellar neuron, the ROI.zip files generated during segmentation by Cellpose 2.0 were opened using the ImageJ ROI.zip macro, which reported the number of segmented cells in each image into a result table that was then exported into a CSV file for quantitative analysis of cell density.

For analysis of each cell population, sampling for cell counting for each cell-specific marker included a minimum of three non-overlapping image planes, from three distinct regions of interest in each of at least three independent sections per rat (3 planes x 3 ROIs x 3 sections = a minimum of 27 images analyzed for each individual rat).

#### Data Summarization

For each cell type of interest, the density of cells identified by colocalization of a neuron-specific marker and DAPI were averaged to calculate the cell density of that type of neuron in lobules 5, 8 and 10 for each animal. These data were then complied to determine the density of each type of neuron in each lobule for WT, HET, and MUT SCA34-KI rats.

#### Statistics and Graphing

Statistical analysis and graphing were performed using GraphPad Prism. Data were analyzed using one-way ANOVA with Tukey’s Post Hoc test. A Bonferroni correction was applied to correct for multiple one-way ANOVAs per cerebellar lobule. Alpha was set to 0.017 and was calculated as 0.05/3 to adjust for the three lobules analyzed for each animal. Data were plotted as violin plots, with the mean cell density (cells/mm^2^) or mean cell size (area in µm^2^) shown as a solid line across the violin; dotted lines show the 25th to 75th percentile range. To show the distribution of the measurements, the mean value for each individual animal in the sample are shown as individual data points (solid for females, open for males).

#### Figure Preparation

To prepare immunolabeling figures, images were imported into Adobe Photoshop. Image scale was calibrated, and adjustments of brightness and contrast were made to highlight image features. Adjustments of brightness and contrast were applied equally to all pixels in the entire image. Graphs were prepared using GraphPad Prism.

## Results

### Identification of Cerebellar Cell Types and Verification of Cell Quantitation

Combinations of cell-specific markers and nuclear labeling were used to identify specific populations of cerebellar neurons to assess their density in WT, HET, and MUT SCA34-KI rat cerebellum. Granule cells (GCs) in the GCL and a population of displaced granule cells located in the inner portion of the ML were identified by immunolabeling for NeuN [41–44]. Molecular Layer Interneurons were identified by labeling for FOX2 [45]. Unipolar brush cells (UBCs), which are particularly concentrated in the posterior of the cerebellum, especially lobules 9 and 10, were identified by labeling for Calretinin [46, 47]. Purkinje cells (PCs), which form a monolayer in the PCL between the GCL and ML, are the sole output neuron of the cerebellar cortex, were identified in histochemically stained sections.

#### Granule Cell Density is Unchanged across Genotypes

**Fig. 3:**
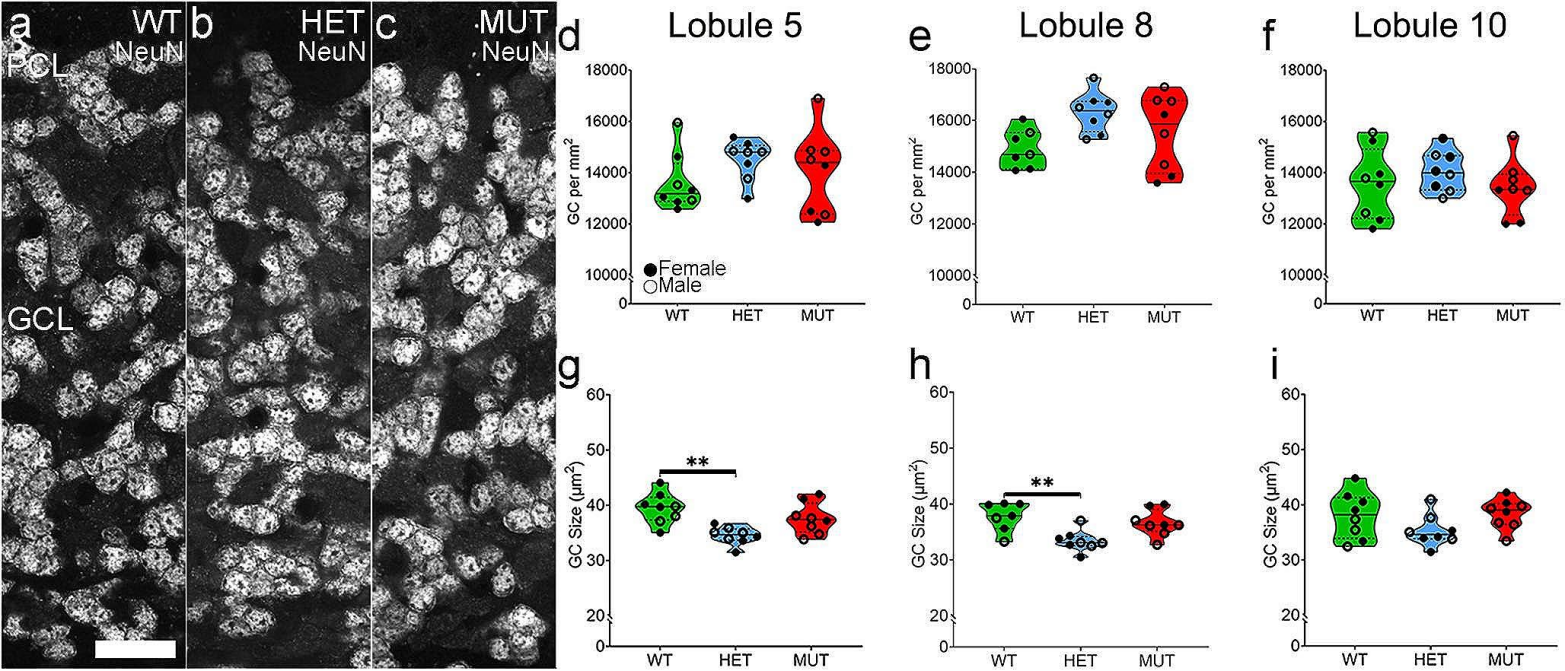
Granule cell density is not affected by the W246G ELOVL4 mutation. **A**-**C**. Micrographs of granule cells labeled for the granule cell-specific marker, NeuN, in the Granule Cell Layer (GCL). **D**-**F**. Granule cell density in lobules 5, 8, and 10. **G**-**I**. Granule cell size in lobules 5, 8, and 10. Each point represents the mean for one animal (WT = 7–8, HET = 8, MUT = 8). One-way ANOVA with Bonferroni correction for multiple analyses was applied, alpha set to 0.017. Scale bar: 20 μm

Granule cells are the most populous neurons of the cerebellum [48], and their loss is characteristic of some ataxic mouse models [49–51]. Granule cells, identified by NeuN labeling (Fig. 3A-C), showed no differences in density across WT, HET and MUT rats across genotypes in any lobule (Fig. 3D-F). Unexpectedly, granule cell soma size was somewhat smaller in lobule 5 and 8 of HET rats compared to WT rats (Fig. 3G-H). Soma size was not affected in MUT rats. There were no significant differences in cell density or size between sexes.

#### Unipolar Brush Cell Density is Unaffected across Genotypes

**Fig. 4:**
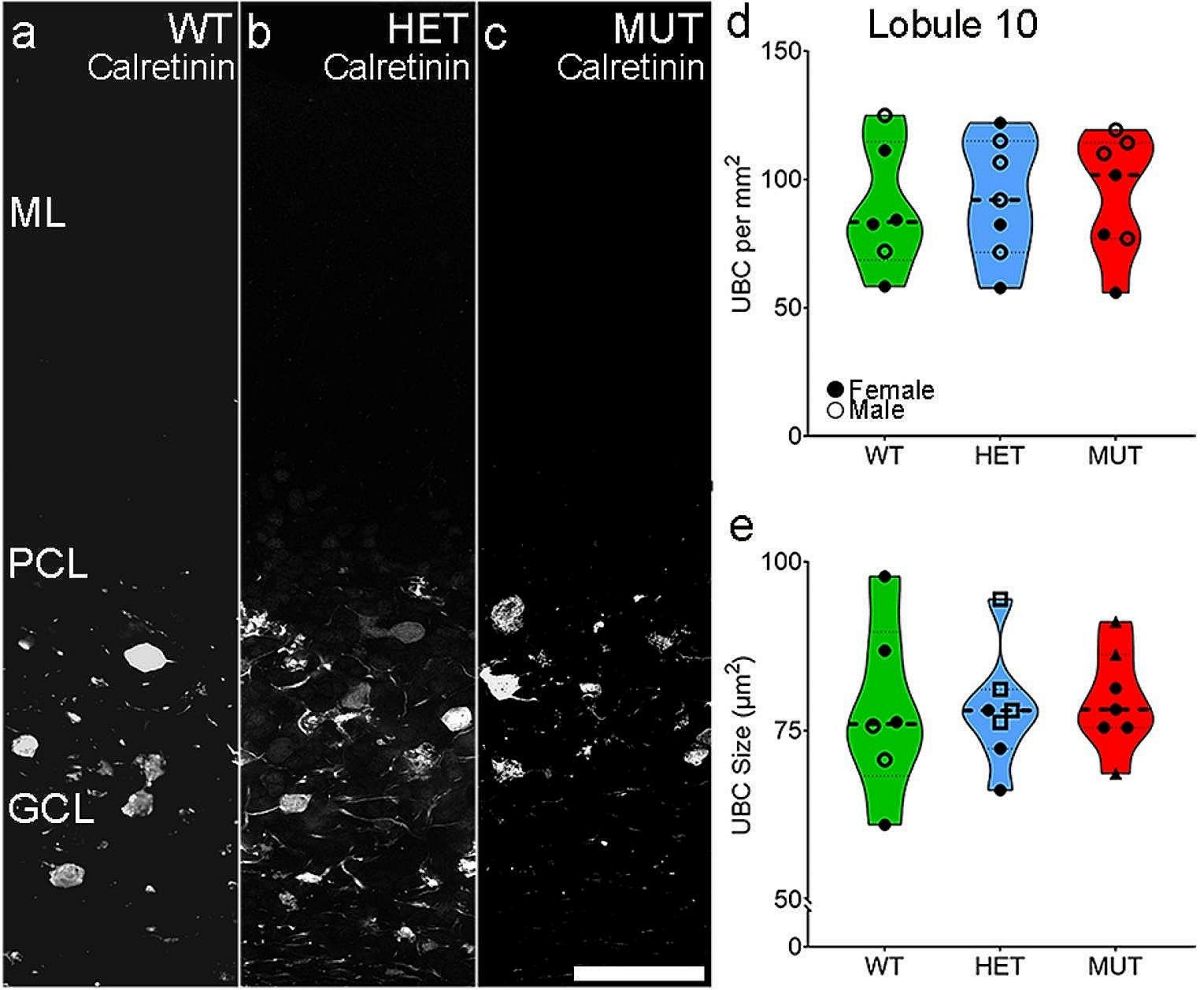
Unipolar brush cell density is not affected by W246G ELOVL4. **A**-**C**. Micrographs of unipolar brush cells identified by Calretinin immunolabeling in lobule 10. Because unipolar brush cell density is extremely low outside of Lobules 9 and 10, unipolar brush cell density was not analyzed in lobules 5 and 8. **D**-**F**. Unipolar brush cell density in Lobule 10. **G**-**I**. Unipolar brush cell size in Lobule 10. Each data point represents the mean for one animal (WT = 6, HET = 7, MUT = 7). One-way ANOVA with Bonferroni correction for multiple analyses was applied, alpha set to 0.017. Scale bar: 50 μm

Unipolar brush cells (UBCs) comprise a small neuronal population, mainly found in the posterior cerebellum. Despite the small total number of UBCs, loss of a subpopulation of UBCs causes ataxia [52]. UBCs showed no differences in either cell density or cell size across WT, HET, and MUT SCA34-KI rats (Fig. 4). Similarly, UBCs showed no sex differences across WT, HET, and MUT SCA34-KI rats.

#### Density of Neurons in the Molecular Layer is Comparable across Genotypes

**Fig. 5:**
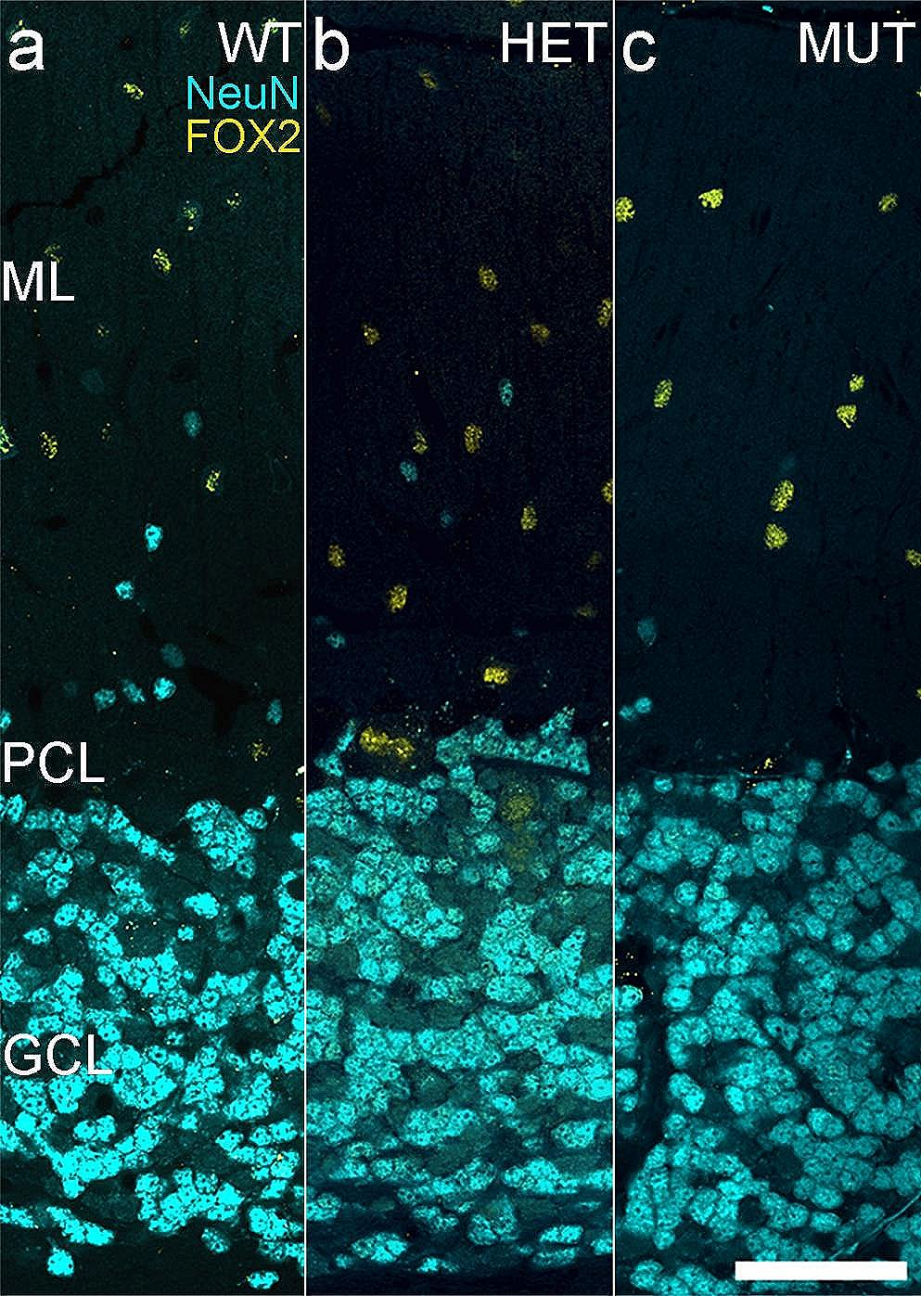
Distinct displaced granule cell and molecular layer interneuron populations are present in the molecular layer. **A**-**C**. Immunolabeling for NeuN (blue) identifies a population of displaced granule cells in the inner portion of the molecular layer as well as granule cells in the granule cell layer. Immunolabeling for FOX2 (yellow) identifies molecular layer interneurons (MLI) distributed throughout the depth of the molecular layer. Displaced granule cells and MLIs are both present in the molecular layer of WT, HET, and MUT rats. (WT = 7, HET = 7, MUT = 8). Scale bar: 50 μm

**Fig. 6:**
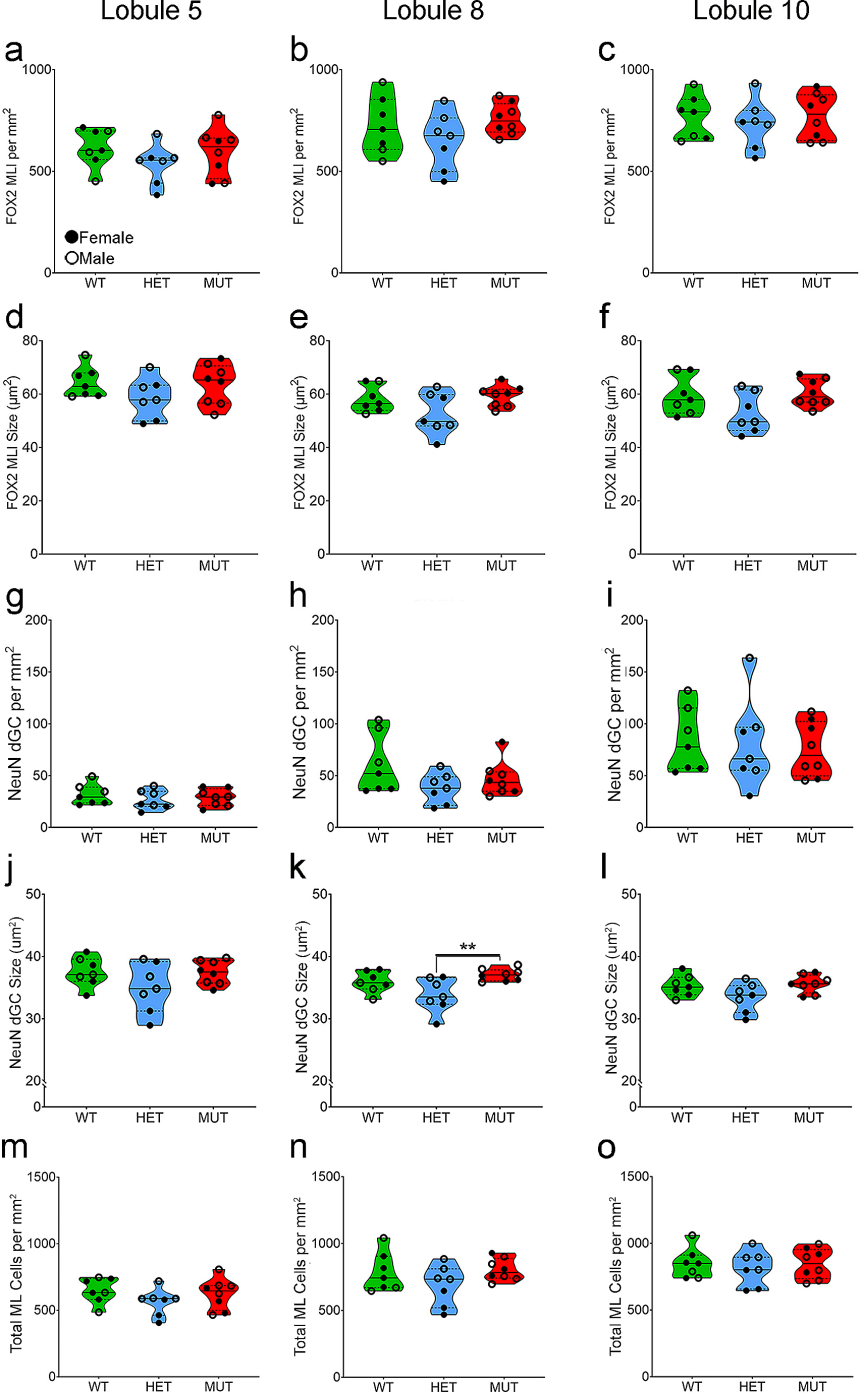
The density of FOX2-positive molecular layer interneurons and displaced granule cells is not affected by the W246G ELOVL4 mutation. **A**-**C**. The density of FOX2-positive molecular layer interneurons is unaffected by W246G ELOVL4. **D**-**F**. The size of FOX2-positive molecular layer interneurons also is unaffected by W246G ELOVL4. **G**-**I**. The density of NeuN-positive displaced granule cells is unaffected by W246G ELOVL4 mutation. **J**-**L**. In contrast, a small but statistically significant difference in the cell body size of NeuN-positive displaced granule cellswas present in the HET and MUT cerebellum. **M**-**O**. The total density of molecular layer neurons, determined by summing the density of Fox2-positive molecular layer interneurons and NeuN-positive displaced granule cells in the molecular layer, was unaffected by W246G ELOVL4. For all graphs, each data point represents the mean for one animal (WT = 7, HET = 7, MUT = 8). One-way ANOVA with Bonferroni correction for multiple analyses was applied, alpha set to 0.017

At least two classes of neurons are present in the ML: MLIs and displaced granule cells. The MLIs are inhibitory neurons and comprise two different subtypes with distinct physiological and gene expression characteristics [53]. The MLIs are distributed across the depth of the ML and can be identified by immunolabeling for FOX2 [45]. The second class of neurons in the ML are a population of displaced granule cells located in the inner portion of the ML [44, 53], which can be identified by labeling for NeuN [42, 43]. Double labeling for FOX2 and NeuN in the ML confirmed that these neurons comprised distinct cell populations (Fig. 5A-C). There was no difference in FOX2-positive MLI density or size across WT, HET, and MUT SCA34-KI rats (Fig. 6A-F). Similarly, the NeuN-positive displaced granule cell population also showed no change in cell density among WT, HET, and MUT SCA34-KI rats (Fig. 6G-I). The size of the NeuN-positive displaced granule cells in the ML showed decreased cell size in HET rats (Fig. 6J-L), similar to the granule cells in the GCL (Fig. 3). No sex differences were noted in density or size of either the FOX2-positive MLIs or the NeuN-positive displaced granule cells in the ML. As a whole, the number of neurons in the ML showed a trend toward increased density from the anterior to posterior cerebellum, and was especially evident for the NeuN-positive displaced granule cells (Fig. 6A-C, G-I).

#### Linear Density of Purkinje Cells is Comparable across Genotypes

**Fig. 7:**
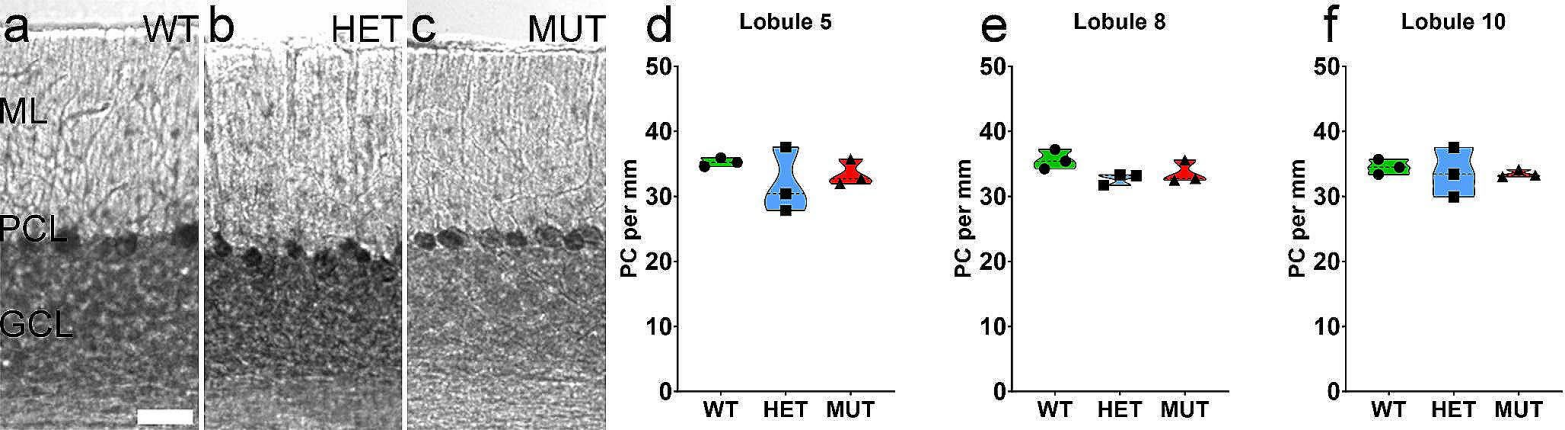
Purkinje cell density is not affected by the W246G ELOVL4 mutation. **A**-**C**. Linear density of Purkinje cells was assessed in cerebellar sections stained with toluidine blue. **D**-**F**. Each point represents the mean for a single female animal (WT = 3, HET = 3, MUT = 3). One-way ANOVA with Bonferroni correction for multiple analyses, alpha set to 0.017. Scale Bar: 20 μm

Purkinje cells are the sole output neuron of the cerebellar cortex [48] and their loss is especially prominent in many types of SCA [18]. Because PCs are very large cells, single confocal image planes often exclude the nucleus of the cell, which would distort analysis of PCs from immunolabeled tissue. However, because Purkinje cells are arranged in a monolayer and morphologically distinct from all other cells in the cerebellum, they are easily quantified from histologically stained tissue Sect. [10]. Therefore, analysis of PC numbers was performed using toluidine blue stained sections of WT, HET, and MUT SCA34-KI rat cerebellum and expressed as a linear density (cells/mm). Purkinje cell linear density was comparable across WT, HET, and MUT SCA34-KI rats (Fig. 7D-F), indicating no deficit in Purkinje cells.

#### Cerebellar Cortex Layer Thickness is Unchanged by Mutant W246G ELOVL4

**Fig. 8:**
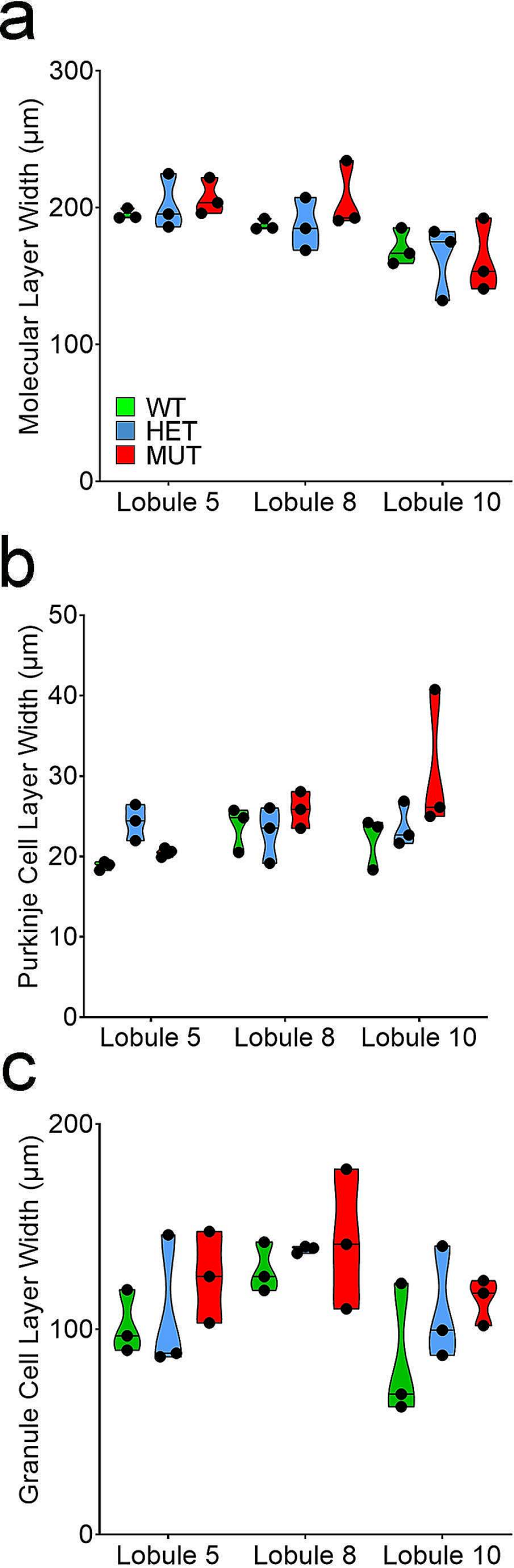
Thickness of cerebellar cortical layers is not affected by the W246G ELOVL4 mutation. Thickness of cerebellar cortical layers were measured at P120 (WT = 3, HET = 3, MUT = 3). (**A**) Molecular Layer. (**B**) Purkinje Cell Layer. (**C**) Granule Cell Layer. Each point represents the mean for a single female animal. One-way ANOVA with Bonferroni correction for multiple analyses, alpha set to 0.017

As an additional assessment of cerebellar structure, the thickness of the GCL, PCL, and ML were measured in lobules 5, 8, and 10 of WT, HET, and MUT SCA34-KI rat cerebellum (Fig. 8A-C). No significant changes in the thickness of any cortical layers were present, consistent with the neuronal population analyses.

## Discussion

Several different single nucleotide polymorphisms in ELOVL4, the fatty acid elongase essential for the synthesis of VLC-SFA, cause SCA34 [19–26]. However, the biological mechanism(s) underlying disease onset and progression remain unclear. Previous studies using the SCA34-KI rat model expressing the W246G ELOVL4 variant that causes human SCA34, showed motor impairment by two months of age, prior to evidence of overt neurodegeneration, suggesting the disease arises from causes other than neurodegeneration [10]. Therefore, we investigated whether W246G ELOVL4 might compromise developmental production or survival of cerebellar neurons.

ELOVL4 is highly expressed in neurogenic regions of the developing brain [7] and has been linked to signaling at the primary cilium [39], an organelle central to regulation of the cell cycle and cell numbers [54, 55]. Further, selective loss of even small populations of cerebellar neurons can cause motor impairment and ataxia [52]. Therefore, we used cell-specific marker labeling and quantitative microscopy approaches to examine whether the early onset of motor impairment in the SCA34-KI rat might arise from neurodevelopmental defects affecting neuronal numbers or the balance of specific neuronal populations in the cerebellum. These analyses suggest that neuronal populations are not substantially altered in the SCA34-KI rat cerebellum at early time points when motor impairment is already present, indicating that other mechanisms underlie the onset of motor impairment.

Granule cells are the largest neuronal population in the cerebellum and comprise several distinct subtypes [48, 56], including a population of displaced granule cells in the inner portion of the ML [43, 57]. Granule cell loss is characteristic of some ataxic mouse models, indicating that aberrant numbers of granule cells can disrupt cerebellar function [49–51].

To evaluate the accuracy of cell selection and quantification, the density of granule cells in the GCL of the vermis in WT rats using the current approach was compared to stereological sampling of semithin Sect. [58]. The current workflow showed granule cell density to be 1.36 × 10^4^ ± 620.0 cells/mm^2^, which is in reasonable agreement with granule cell density reported in the previous stereological analysis, 1.19 × 10^4^ cells/mm^2^ [58]. It is important to note that estimation of cell populations by sampling of two-dimensional image planes as performed in this report, potentially could introduce some level of uncertainty due to the three-dimensional nature of cell distribution in the tissue sampled.

Purkinje cells are the sole output neuron of the cerebellar cortex and commonly degenerate in SCA [18, 59]. The linear density of Purkinje cells in WT, HET and MUT SCA34-KI rat cerebellum showed no differences in any lobule by P120, well after motor impairment is readily detectable by P60 [12]. Thus, aberrant numbers or survival of Purkinje cells cannot explain the onset of motor impairment in the SCA34-KI rat. A previous stereological report estimated a GC: PC ratio of 274 GCs/PC [58]. Our analysis of WT rat cerebellum yielded an estimated GC: PC ratio of 386 GCs/PC, which is somewhat higher, but still in approximate agreement.

The MLIs provide inhibitory input to Purkinje cells [60], and comprise two distinct functional populations based on physiological and gene expression characteristics that are independent of classical basket cell or stellate cell morphology [53]. The density of MLIs did not differ across genotypes or sex, indicating that altered MLI populations are not responsible for motor impairment in SCA34.

Unipolar brush cells comprise a very small proportion of cerebellar neurons, receiving input from extrinsic mossy fibers and providing glutamatergic input to granule cells [61]. They are concentrated mainly in the posterior cerebellum, especially lobule 10, and are particularly important to vestibular function [62]. Although UBCs comprise only a tiny proportion of cerebellar neurons, they are functionally critical as their absence causes ataxia [52]. There were no differences in UBC numbers or size across genotypes or sex indicating that aberrant numbers of UBCs is not responsible for the motor impairment in HET and WT SCA34-KI rats.

The cerebellum houses additional types of inhibitory interneurons that comprise relatively small cell populations including, Golgi, Lugaro, globular, and candelabrum cells [63]. Unique immunohistochemical markers to definitively distinguish these different cell classes have not been described to date, precluding direct assessment of these cells in the current study. Given that mutant W246G ELOVL4 did not affect the numbers of other cerebellar cells, it is likely that the size of these cell populations are altered in the SCA34-KI rat cerebellum although that possibility cannot be entirely eliminated.

Consistent with the analyses of specific cell populations, the thickness of the layers of the cerebellar cortex did not differ across WT, HET, and MUT SCA34-KI rats at P120. These findings indicate that large scale neurodegeneration was absent in the HET or MUT SCA34-KI rat cerebellum at this age. These findings are consistent with a previous report showing no significant differences in cortical layer widths in WT, HET, and MUT SCA34-KI rat cerebellum at 3 and 6 months of age [12]. Taken together, these data indicate that the early onset of motor impairment in HET and MUT SCA34-KI rats does not stem from neurodevelopmental errors in neuronal production leading to the absence of specific types of cerebellar neurons, an imbalance among neuronal populations, or from neurodegeneration.

In the current study, granule cells and displaced granule cells in the HET SCA34-KI rat showed small, but statistically significant reductions in cell size. The meaning of this finding is unclear, but it is interesting that differences in cell size were noted only in granule cell populations. It is also interesting that ELOVL4 has been linked to ciliary signaling [39], which regulates cell cycle but also can affect cell size [64]. Further investigation will be required to elucidate the role of ELOVL4 in cell size regulation.

Together, these experiments indicate that neuronal populations are not perturbed in the cerebellum of the SCA34-KI rat, even at timepoints when significant motor impairment is already present [12], suggesting that SCA34 does not arise from aberrant generation of cerebellar neurons or directly from neurodegeneration. Given the current results, the best candidate mechanism for the initial motor impairment observed in SCA34-KI HET and MUT rats is synaptic dysfunction [12]. Previous studies performed using SCA34-KI rat retina and HEK cells transduced with disease-causing *ELOVL4* variants indicate that W246G ELOVL4 and other SCA34-causing variants of ELOVL4 selectively impair synthesis of VLC-SFA compared to wildtype ELOVL4 [24, 37, 38]. This leads to impairment of synaptic transmission by cerebellar neurons in the SCA34-KI rat [12]. The SCA34-KI rat also shows aberrant cerebellar synaptic function [12]. Long-term potentiation and long-term depression, which are critical to normal cerebellar function, are severely impaired in the SCA-34-KI rat cerebellum, and synaptic network activity in the SCA34-KI rat cerebellum is also aberrant [12]. Patch-clamp studies in cerebellar slices from WT and MUT SCA34-KI rats show abnormal presynaptic release at parallel fiber, climbing fiber, and inhibitory synaptic inputs to Purkinje cells [15]. Mild impairments in synaptic transmission by retinal photoreceptors also are present in the SCA34-KI rat [37]. These findings are consistent with aberrant synaptic release kinetics and intractable seizures reported in a mouse model of neuroichthyosis arising from the absence of ELOVL4 function in the brain [13]. That study also showed addition of exogenous VLC-SFA to cultured neurons lacking functional ELOVL4 rescued synaptic function [13].

## Conclusion/Summary

These studies in the SCA34-KI rat model indicate that the W246G ELOVL4 mutation that causes Spinocerebellar Ataxia 34 does not alter the production or survival of the major populations of cerebellar neurons at early ages. These results suggest that the early impairment of motor function noted in the SCA34-KI rat model of human SCA34, initially arises from synapse-specific anomalies in neurotransmission and synaptic plasticity, rather than neurodevelopmental errors affecting the production or survival of cerebellar neurons.

## Data Availability

All data generated or analyzed during this study are included in this published article [and its supplementary information files]. Additional information needed on the datasets used and/or analyzed during the current study are available from the corresponding author on reasonable request.
